# Environment-sensitive behavior of fluorescent molecular rotors

**DOI:** 10.1186/1754-1611-4-11

**Published:** 2010-09-15

**Authors:** Mark A Haidekker, Emmanuel A Theodorakis

**Affiliations:** 1 Faculty of Engineering, 597 D.W. Brooks Drive, University of Georgia, Athens, GA 30602, USA; 2 Department of Chemistry and Biochemistry, University of California, San Diego, 9500 Gilman Drive, San Diego, CA 92093, USA

## Abstract

Molecular rotors are a group of fluorescent molecules that form twisted intramolecular charge transfer (TICT) states upon photoexcitation. When intramolecular twisting occurs, the molecular rotor returns to the ground state either by emission of a red-shifted emission band or by nonradiative relaxation. The emission properties are strongly solvent-dependent, and the solvent viscosity is the primary determinant of the fluorescent quantum yield from the planar (non-twisted) conformation. This viscosity-sensitive behavior gives rise to applications in, for example, fluid mechanics, polymer chemistry, cell physiology, and the food sciences. However, the relationship between bulk viscosity and the molecular-scale interaction of a molecular rotor with its environment are not fully understood. This review presents the pertinent theories of the rotor-solvent interaction on the molecular level and how this interaction leads to the viscosity-sensitive behavior. Furthermore, current applications of molecular rotors as microviscosity sensors are reviewed, and engineering aspects are presented on how measurement accuracy and precision can be improved.

## Introduction

**Figure 1:**
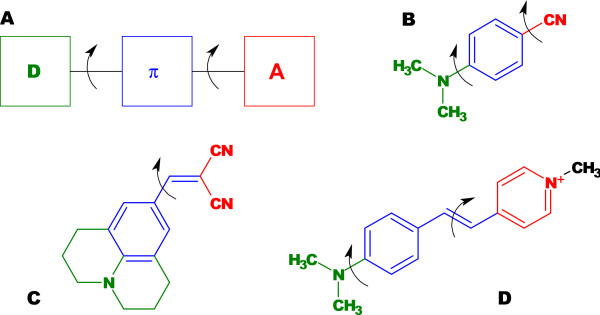
**General structure of a molecular rotor (*A*), highlighting the electron donating subunit (green), the electron accepting subunit (red), and the spacer unit (blue), which spatially separates the donor and acceptor groups**. Typical representatives are aniline nitriles, such as 1,4-dimethylamino benzonitrile (DMABN, *B*), julolidine malononitriles, such as 9-(dicyanovinyl) julolidine (DCVJ, *C*), and stilbenes, such as p-(dimethylamino) stilbazolium (p-DASPMI, *D*). The arrows indicate bonds around which intramolecular rotation can take place.

The term *molecular rotor *is commonly used to describe a fluorescent molecule that has the ability to undergo an intramolecular twisting motion in the fluorescent excited state. Typically, a molecular rotor consists of three subunits, an electron donor unit, an electron acceptor unit, and an electron-rich spacer unit that is composed of a network of alternative single and double bonds. This network brings the donor and acceptor units in conjugation, thus facilitating electron movement between this pair, but it ensures minimum overlap of the electron donor and electron acceptor orbitals [1]. In this configuration, the molecule responds to photoexcitation with an intramolecular charge transfer from the donor to the acceptor unit. Whereas the three subgroups assume a planar or near-planar configuration in the ground state, electrostatic forces induce an intramolecular twisting motion of the sub-groups relative to each other [2]. The molecule enters a nonplanar (twisted) state with a lower excited-state energy, and relaxation from the twisted state is associated with either a red-shifted fluorescence emission or nonfluorescent relaxation, depending on the molecular structure [3,4]. The basic structure of a molecular rotor, together with several typical examples, is shown in Figure 1. Moreover, several chemical classes of molecular rotors exist [5], which are listed in Table 1 together with photophysical characteristics of typical representatives [6-9]. Other fluorophores exist which show predominantly polarity-sensitive behavior that has been attributed to formation of twisted states, but they are much less well-explored than the classes listed in Table 1. Examples are Nile Red [10,11] and 8-(phenylamino)-1-naphthalenesulfonate (ANS) [12]. A meso-substituted form of dipyrrometheneboron difluoride (BODIPY) has also been hypothesized to form twisted states [13]. However, chemical computations by Kee *et al*. [13] present a very atypical picture where a planar configuration (0° or 180°) of a 5-aryl-substituted dipyrrin is energetically preferred in the excited state, whereas the lowest-energy angle is 55° in the ground state, i.e., only 35° from perpendicularity. A low degree of rotation would correspond to a low sensitivity towards the environment. For these classes of dyes, further research is needed to understand the specific fluorophore-solvent interactions and the role of segmental mobility in the possible formation of TICT states.

**Table 1:** Overview of the most important groups of molecular rotors with the key spectral properties of one representative example.

Group	Representative example	Peak excitation (approximate)	Peak emission (LE, TICT)	Reference
Benzonitrile-based fluorophores	DMABN	290 nm	342 nm, 460 nm	[6]

Benzylidene malononitriles	DCVJ	489 nm	505 nm^1^	[7]

Stilbenes	p-DASPMI	470 nm	560 nm^1^	[8]

Arylmethine dyes	Crystal violet	590 nm	630 nm^1^	[9]

^1^indicates single-band emission from the LE conformation with nonradiative relaxation from the TICT conformation.

Relaxation from the TICT state occurs in one of two ways (see Table 1): In the case of DMABN, the *S*_1 _- *S*_0 _energy gap in the twisted state is large enough to allow photon emission when the molecule returns to the ground state in a twisted conformation. Such a molecule exhibits a distinct second emission band that is red-shifted from the LE fluorescence. DMABN, for example, has a twisted-state energy gap that is approximately 30% lower than the LE energy gap, and relaxation from both LE and twisted conformation leads to photon emission. Conversely, when the TICT energy gap is much smaller than the LE energy gap, nonradiative relaxation occurs from the TICT conformation. In the example of DCVJ, the twisted-state *S*_1 _- *S*_0 _energy gap is three times smaller than the LE energy gap [4]. Fluorophores of this class exhibit only a single emission band.

The most notable feature of molecular rotors is the dependency of the twisted state formation rate on the local microenvironment, predominantly the microviscosity of the solvent. In the case of molecular rotors that emit from the twisted state with a red-shifted emission band, steric hindrance of the twisted-state formation in higher-viscosity solvents changes the emission in favor of the shorter-wavelength emission from the planar state [14]. In the case of molecular rotors that exhibit nonradiative relaxation from the twisted state, the fluorescent quantum yield increases in higher-viscosity solvents [15]. The molecular and photophysical phenomena that govern this behavior are explained after the next section.

## Biological and Chemical Applications of Molecular Rotors

Bulk viscosity measurement of fluids advertises itself as a possible application. Viscosity changes of protein-containing biofluids, i.e., blood plasma and interstitial fluid, have been linked to various diseases [16] that are mostly associated with altered protein levels. Examples include infections and infarction [17], hypertension [18], diabetes [19], atherosclerosis [20], and normal aging [21]. Furthermore, one of the adverse effects of smoking is elevated plasma viscosity [22], which may be the link between cigarette consumption and cardiovascular disease. The viscosity of lymphatic fluid is directly linked to blood plasma viscosity, because the lymphatic system captures fluid and protein that diffused into the tissue and returns it to the vascular system. Lymphatic fluid viscosity is increased, for example, in conjunction with breast cancer treatment [23], and viscosity changes alter lymphatic fluid circulation during acute shock [24].

Some progress has been made in providing proof-of-principle for this application [25] and in demonstrating that measurement precision of an optical method based on fluorescent molecular rotors is comparable to that of conventional mechanical rheometers [26]. However, few studies exist where bulk viscosity of fluids has been measured with fluorescent molecular rotors. A likely reason is the wide availability of established mechanical rheometers [27]. Moreover, fluorescence-based methods are confounded by the optical properties of the liquid, and correction methods are still under investigation [28]. On the other hand, mechanical rheometers are time-consuming due to single measurements requiring measurement times in the range of minutes, they require scrupulous cleaning and are limited to bulk volumes. These disadvantages make fluorescence-based viscsoity measurements an attractive proposition, most notably due to their considerable speed advantage over mechanical methods [26].

A popular application of molecular rotors is real-time monitoring of aggregation and polymerization processes. Loutfy and Teegarden [29] demonstrated that the emission intensity of DCVJ, but not its peak emission wavelength, strongly depend on the tacticity of PMMA macromolecules: When DCVJ was embedded in PMMA films, it exhibited a quantum yield of 0.015 - 0.020 in syndiotactic PMMA and a slightly higher quantum yield of 0.018 - 0.025 in atactic PMMA. Quantum yield was markedly increased to 0.036 - 0.049 in isotactic PMMA, leading to the conclusion that the flexibility of isotactic chains is lower than that of atactic and syndiotactic chains. Loutfy [30] also demonstrated that the quantum yield of a molecular rotor related to DCVJ increased in polystyrene samples with increasing molecular weight. Similarly, Zhu *et al*. [31] found that emission of the molecular rotor FCVJ (a hydrophobic ester of (2-carboxy-2-cyanovinyl)-julolidine [7]) is strongly dependent on the chain entanglement in macromolecules - FCVJ fluorescence intensity accurately reported whether polypropylene oxide melts were in the Rouse or the reptation regime.

Molecular rotors have been used to report protein aggregation and protein conformational changes. Hawe *et al*. [32] showed that heat stressing of immunoglobulin-polysorbate 4 preparations changed the balance of DCVJ and (2-carboxy-2-cyanovinyl)-julolidine towards preferentially binding to polysorbate and thus decreasing their quantum yields. Similar observations were not possible with Nile Red [32]. Not only protein aggregation within the rotor's solvent causes the fluorescence to shift towards emission from the planar state, but also binding of the molecular rotor to a protein. In this context, molecular rotors become fluorescent probes for protein conformational changes and protein assembly. Kung and Reed [33] have shown that DCVJ binds to tubulin, thereby increasing DCVJ quantum yield. DCVJ further increased its fluorescence emission intensity when tubulin aggregated as tubules over tubulin aggregating as sheets [33]. In this study, the peak emission wavelength of DCVJ remained widely constant, indicating that viscosity and polarity do not cause a significant solvatochromic shift, an observation that was later confirmed by our group [34]. Sawada *et al*. [35] used a molecular rotor related to DCVJ to noncovalently bind to actin, and observed that the transition from G-actin to F-actin was accompanied by a strong intensity increase form the molecular rotor reporter. Lindgren *et al*. [36] examined the folding kinetics of transthyretin, a protein known to misfold and form amyloid deposits in peripheral nerves. The authors found that the molecular rotors DCVJ and thioflavin T preferentially bind to misfolded transthyretin and allow to specifically monitor the formation dynamics of pathogenic transthyretin aggregates.

The characterization of cyclodextrins is another representative area where the ability of molecular rotors to report the properties of the microenvironment plays a key role [37,38]. Cyclodextrins have a hydrophobic core that can be used to deliver hydrophobic compounds to aqueous environments (e.g., drug delivery) [39]. To optimize cyclodextrins for a particular purpose, the nature of the core region needs to be explored. Dual-emission molecular rotors, such as DMABN, are ideally suited for this task, because emission from the twisted state reports hydrophobicity, whereas emission from the planar state reports on the restricted environment of the core.

Another area where molecular rotors found widespread application is the examination 5 of phospholipid bilayers and cell membranes [40-43]. Lukac [44], for example, examined several phospholipids that were stained with molecular rotor reporters under varying temperature conditions and found a change in the temperature-dependent behavior: When the phospholipid transitioned from the gel- to the liquid-crystal phase, a blue-shift of the emission was observed when the rotor molecules moved towards the more hydrophobic center of the bilayer as the bilayer "melted" at higher temperature. Moreover, Lukac was able to deduce an apparent bilayer microviscosity for the individual phospholipids. Following the same line of investigation, Nipper *et al*. [45] demonstrated that microviscosity, determined through molecular rotor fluorescence, correlates highly with the viscosity determined through fluorescence recovery after photobleaching (FRAP). FRAP is a microscopy method where fluorophore diffusivity in a phospholipid membrane can be determined, thus allowing to estimate microviscosity.

Whereas many fluorescent probes in biology predominantly offer qualitative information, the promise of molecular rotor fluorescence is the *quantitative *nature of the fluorescent response. In fact, fluorescence emission of molecular rotors can be used to determine the microviscosity of the environment with the same level of rigor as two established methods, FRAP [46] and fluorescence anisotropy [47]. All three methods are based on diffusion. FRAP is governed by lateral diffusion of a fluorophore into a region where the dye has been destroyed by intense light. Fluorescence anisotropy is governed by the rotational diffusion of a dye that has been excited by polarized light, where rotational diffusion leads to depolarization of the emission light. Rotational diffusion governs the propensity of a molecular rotor to form twisted states and therefore relates diffusivity to fluorescence quantum yield. In this respect, molecular rotors report diffusivity similar to anisotropy probes. However, the dominating factor in molecular rotor emission is the rotation of one segment relative to the other. The segment (such as the dimethylamino group or the dicyanovinyl segment) is generally very small and enjoys greater freedom of rotation than a typical anisotropy probe, such as 1,6-diphenyl-1,3,5-hexatriene (DPH). The relationship between viscosity, rotational diffusivity, and intramolecular rotation makes molecular rotors attractive reporters for the microenvironment, because the sensing of the diffusivity - and with it, microviscosity - can be reduced to simple and rapid spectroscopic intensity measurements [16].

## Photophysical Principles of Molecular Rotors

**Figure 2:**
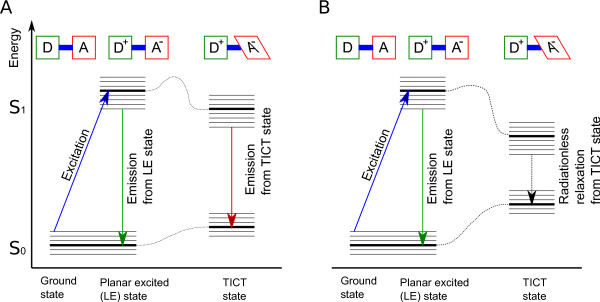
**Extended Jabłonski diagram for molecular rotors**. Like conventional fluorophores, a molecular rotor is elevated from the ground state (*S*_0_) to the energetically higher first excited state (*S*_1_) by photon absorption. Different vibrational states (indicated by parallel lines) cause some energy loss, and emission from the LE state occurs at a longer wavelength than the excitation (Stokes shift). For molecular rotors, the Jabłonski diagram needs to be extended, because the excited-state energy is lower in the TICT state, whereas the ground-state energy is higher in the TICT state than in the LE state. Therefore, the *S*_1 _- *S*_0 _energy gap is lower in the TICT state with a correspondingly lower relaxation energy. In the case of moelcular rotors with dual-band emission (such as DMABN), the TICT energy gap is slightly smaller than the LE energy gap (A). If the TICT energy gap is much smaller than the LE energy gap, for example in DCVJ, emission from the TICT state occurs without photon emission (B). This diagram does not reflect intersystem crossing, because triplet states do not play any significant role in the fluorescence of molecular rotors.

A fluorescent intramolecular charge-transfer (ICT) complex is elevated to a higher energy level after photoabsorption by charge separation, that is, an electron is transferred from the donor unit to the acceptor unit. With reference to Figure 1A, the molecule assumes the excited-state configuration *D*^+ ^- *π *- *A*^-^. The charge separation is associated with an increased dipole moment. In the example of DMABN, the ground-state dipole moment is approximately 6 Debye (1 Debye [D] ≈ 3.336·10^-23^*C*·*m*), and the excited-state dipole moments have been found to be approximately 10 Debye in the planar conformation and between 19 and 22 Debye, depending on the solvent, in the TICT (twisted intramolecular charge transfer) conformation [48]. Polar solvent molecules orient themselves along the fluorophore dipole by aligning their electric fields. Upon relaxation, the solvent molecules return to the ground-state orientation. As a consequence, the fluorophore exhibits a bathochromic shift that reflects the energy expended for the reorientation of the solvent molecules. The magnitude of this effect depends on the solvent polarity, that is, its dielectric constant [49]. Molecular rotors typically exhibit stronger solvatochromism in the twisted-state emission band than in the planar locally excited (LE) emission band. Furthermore, both the ground-state and the excited-state energy levels depend on the degree of intramolecular rotation [50-52]. The Jabłonski diagram of the energy states can be extended to include the TICT-state energy levels (Figure 2). In the ground state, a planar conformation is energetically preferred, whereas the twisted conformation is the preferred conformation in the excited state. Therefore, the excited molecule rapidly assumes the twisted-state conformation. Viscous solvents are known to increase the energy barrier between the LE and TICT states (indicated by a dashed line in Figure 2), and emission shifts in favor of the LE band. Figure 2 does not include triplet states, because intersystem crossing plays a negligible role in the spectral emission of molecular rotors. The TICT formation rate emerges as the dominant factor that determines the relative intensity of the two emission bands in the case of molecular rotors with dual emission and the overall quantum yield in the case of molecular rotors with radiationless relaxation from the TICT state.

## Interactions of Molecular Rotors with the Environment

The solvent directly influences the TICT formation rate. Polar solvents are known to stabilize the TICT state [48] and thus increase relaxation from the TICT state. The polarity of molecules is associated with the ability to form hydrogen bonds, and hydrogen bond formation between molecular rotors and the solvent has been found to increase TICT formation rate [53]. With respect to the applications that were presented earlier, the most important rotor-solvent interaction is the sensitivity towards the solvent's viscosity. In viscosity-sensing applications, molecular rotors with a single emission band are most widely used, because emission from the LE state is relatively insensitive towards the solvent polarity [4,34,41]. LE-state quantum yield *ϕ_F _*and bulk viscosity *η *follow a power-law relationship that is widely referred to as the Förster-Hoffmann equation,

(1) $$log\phi_{F}=C+x\;\cdot \;log\;\eta$$

where *C *and *x *are solvent- and dye-dependent constants. This relationship has been experimentally shown to be valid in a wide range of viscosities and in both polar and nonpolar fluids [15,34,40,54,55], although deviations exist particularly in the low-viscosity regime that need additional interpretation. Equation 1 has become so popular that in some instances the existence of this power-law relationship has been used to purport TICT behavior of specific molecules [56-59].

Equation 1 holds the prisomise of a quantitative relationship between viscosity and quantum yield. Steady-state emission intensity is proportional to the quantum yield, and the excited-state lifetime *τ *is related to the quantum yield through

(2) $$\phi_{F}=\frac{\tau }{\tau_{0}}$$

where *τ*_0 _is the natural lifetime of the fluorophore. To understand the relationship in Equation 1 and the significance of the constants *C *and *x*, it is necessary to closely examine the relationship between bulk viscosity, molecular-scale interaction of the molecular rotor with the solvent, and the fluorescence quantum yield.

## Quantitative Influence of Diffusion on Tict Behavior

Currently accepted theories of rotor-solvent interaction center on diffusion. Two derivations of Equation 1 are based on Debye-Stokes-Einstein diffusion and free-volume diffusion, respectively. Förster and Hoffmann [9] provided a rigorous derivation based on classical mechanics and the assumption of Debye-Stokes-Einstein (DSE) diffusion. In their ground-breaking work on triphenylamine dyes, Förster and Hoffmann postulated that each aniline group acts as a nanoscale ellipsoid that obeys the second-order differential equation of rotational motion,

(3) $$\theta \frac{d^{2}\varphi }{dt^{2}}+\kappa \frac{d\varphi }{dt}+\alpha (\varphi -\varphi_{0})=0$$

where *φ *is the rotational angle of the aniline group with respect to the ground-state equilibrium position *φ*_0_, *θ *is the rotational inertia of the aniline group, and *α *reflects the electrostatic force that returns the aniline group to its equilibrium position. The microfriction *κ *is linked to bulk viscosity *η *through the DSE diffusion model,

(4) $$\kappa =8\pi \;r^{3}\eta$$

where *r *is the effective radius of the aniline group. Under the assumption of strong damping (more precisely, *κ*^2 ^≫ 4*κθ*), a twisted aniline group returns to the ground-state equilibrium in an exponential-decay fashion with a decay constant of *κ*/*α*. Förster and Hoffmann define a function *B*(*φ*) as *B*(*φ*) = *β*(*φ*-*φ*_0_)^2 ^that describes the rate of deactivation processes through conformational changes, with *β *being a proportionality constant. With this definition, a differential equation can be found that governs the probability *ρ*(*t*) that the molecule is in the excited state:

(5) $$\begin{array}{r} -\frac{d\rho (t)}{dt}=\left(\frac{1}{\tau_{s}}+B(\varphi )\right)\rho (t)\\ =\left(\frac{1}{\tau_{s}}+\beta \delta {\left(1-e^{-t/(\kappa /\alpha )}\right)}^{2}\right)\rho (t) \end{array}$$

Here, *δ *is the angular difference between the lowest-energy conformations in the excited and ground states and *τ_s _*is the lifetime of the fluorophore in the absence of rotational relaxation events. The quantum yield *ϕ_F _*can be obtained by integrating the excited-state probability:

(6) $$\phi_{F}=\frac{1}{\tau_{0}}\int_{0}^{\infty }\rho (t)dt$$

Although not defined in the original manuscript [9], *τ*_0 _can be assumed to be the natural lifetime, that is, the lifetime of the fluorophore in the absence of any nonradiative deactivation processes as opposed to *τ_s_*, which is the lifetime in the absence of only rotational deactivation processes. According to Förster and Hoffmann, typically *τ_s_*/*τ*_0 _≈ 0.5 for this class of molecular rotors [9]. To simplify the solution of Equation 5 and its subsequent integration (Equation 6), Förster and Hoffmann examined three special cases. The first case (Equation 7) emerges for low viscosities where the quantum yield reaches a solvent-independent minimum:

(7) $$\phi_{F,min}=\frac{1}{\beta \tau_{0}\delta^{2}}$$

The second case occurs in solvents of very high viscosity, where radiative relaxation dominates with negligible rotational relaxation, and the quantum yield can be approximated by Equation 8:

(8) $$\phi_{F,max}=\frac{\tau_{s}}{\tau_{0}}\left(1-\frac{6\sigma^{2}}{\eta^{2}}\right)$$

In Equation 8, *σ *is a dye-dependent constant that contains all viscosity-independent variables and has units of viscosity:

(9) $$\sigma ={\left(\frac{\alpha^{2}\beta \delta^{2}\tau_{s}^{3}}{192\pi^{2}r^{6}}\right)}^{\frac{1}{2}}$$

The most important case, the third case, is found for intermediate viscosities *η *≪ *σ*, when the solution of Equation 6 simplifies to Equation 10:

(10) $$\phi_{F}=0.893\;\cdot \;\frac{\tau_{s}}{\tau_{0}}{\left(\frac{\eta }{\sigma }\right)}^{\frac{2}{3}}$$

For crystal violet, a triphenylmethane dye, *σ *≈ 100 Pa s can be found. The remaining constants can be combined into one constant $\hat{C}$, yielding Equation 11, which is the non-logarithmic form of Equation 1 with an exponent *x *≡ 2/3 as the result of an integration step:

(11) $$\phi_{F}=\hat{C}\cdot \eta^{2/3}$$

Other quantitative treatments of the viscosity-sensitive behavior are based on the premise that the intramolecular reorientation rate *k_or _*depends on rotational diffusivity, more precisely, *k_or _*∝ *D*. A fluorophore's quantum yield *ϕ_F _*is defined as the radiative relaxation rate *k_R _*relative to the total relaxation rate *k_R _*+ *k_NR _*(Equation 12):

(12) $$\phi_{F}=\frac{k_{R}}{k_{R}+k_{NR}}\approx \frac{k_{R}}{k_{or}}$$

The approximation is valid for molecular rotors, because the intramolecular reorientation rate *k_or _*is the dominant nonradiative relaxation pathway, and *k_or _*≫ *k_R_*. Furthermore, *k_R _*is a viscosity-independent dye constant. DSE diffusion stipulates that the rotational diffusion constant *D *is inversely proportional to viscosity (Equation 13):

(13) $$D=\frac{1}{6V\;s\;g}\;\cdot \;\frac{k_{Β}T}{\eta }$$

Here, *V *is the effective volume of the molecule, *s *reflects a boundary condition (*s *= 1 for a stick condition and *s *< 1 for a slip condition), *g *is a shape factor, *k_B _*is Boltzmann's constant, and *T *is the temperature. Vogel and Rettig [60] define a driving force *F *, which is the force constant of the harmonic twist potential in triphenylmethane dyes, such that

(14) $$k_{or}=\frac{2F}{\zeta_{S}}$$

where *ζ_S _*is the Stokes friction coefficient, defined as *ζ_S _*= 6*V η*, implicitly setting *s *= 1 and *g *= 1. Vogel and Rettig now argue that the product of viscosity and rotational reorientation rate would be constant under the DSE theory (Equation 15) [60].

(15) $$k_{or}\eta =\frac{F}{3V}$$

Experimental evidence invalidates Equation 15, because the product *k_or_η *increases strongly with decreasing temperature. To explain the deviation from DSE theory, Vogel and Rettig use the microfriction model introduced by Gierer and Wirtz [61]. These authors extend DSE theory by accounting for the finite thickness of molecular layers that surround the fluorophore. By solving the equation of rotational motion for a spherical molecule of radius *r_M _*surrounded by finite layers of spherical solvent molecules with a radius *r_S _*, Gierer and Wirtz obtain a corrected microfriction coefficient *ζ_Micro _*that is related to the DSE macrofriction coefficient *ζ_Macro _*through Equation 16 [61]:

(16) $$\zeta_{Micro}=\zeta_{Macro}\cdot {\left(\frac{6r_{S}}{r_{M}}+\frac{1}{{\left(1+r_{S}/r_{M}\right)}^{3}}\right)}^{-1}$$

Vogel and Rettig interpret this result as a superposition of Stokes diffusional freedom 1/*ζ_S _*and free-volume diffusional freedom 1/*ζ_FV _*(Equation 17), where diffusion is facilitated by void spaces between solvent and solute.

(17) $$\frac{1}{\zeta_{Micro}}=\frac{1}{\zeta_{S}}+\frac{1}{\zeta_{FV}}$$

Viscosity decreases with increasing temperature. A commonly-used model is the Arrhenius function

(18) $$\eta =\eta_{0}\cdot exp\left(\frac{E_{A}}{k_{B}T}\right)$$

where *η*_0 _is a material constant, *E_A _*is an apparent activation energy, *k_B _*is Boltzmann's constant, and *T *is the absolute temperature. Since viscosity is assumed to be proportional to the friction coefficient *ζ*, the apparent microviscosity *η_Micro _*of the solvent, which is reported by the molecular rotor, would be smaller than the DSE macroviscosity. More specifically, the apparent microviscosity can be described as the superposition of two Arrhenius terms (Equation 19),

(19) $$\begin{array}{c} \eta_{Micro}=a\cdot exp\left(-\frac{E_{\eta }}{k_{B}T}\right)\\ +b\cdot exp\left(-\frac{E_{FV}}{k_{B}T}\right) \end{array}$$

where *a *and *b *are related to the material constant *η*_0_, and *E_η _*and *E_FV _*are the apparent activation energies for DSE macroviscosity and the free-volume viscosity term, respectively. After some arithmetic manipulation, Vogel and Rettig arrive at an extension of Equation 15, namely, *k_or_η *= *A *+ *B η^x^*, where *A *corresponds to the term *F*/3*V *in Equation 15 and *x *reflects the relative contribution from Stokes and free-volume diffusion and is not identical to the exponent *x *in Equation 1. With *A *and *B *being experimental constants, this model was found to represent a good fit of experimental data [60]. To obtain an equation similar to Equation 11, *k_or _*can be substituted in Equation 12, leading to Equation 20, which is an alternative model to Equation 1:

(20) $$\phi_{F}=\frac{k_{R}\;\eta }{A+B\;\eta^{x}}$$

Free volume was also recognized as an important determinant of a molecular rotor's quantum yield by Loutfy and coworkers [62-64]. The treatment by Loutfy *et al*. is based on Doolittle's [65] empirical relationship between viscosity and free volume, Equation 21,

(21) $$\eta =A\cdot exp\left(B\frac{v_{o}}{v_{f}}\right)$$

where *A *and *B *are empirical, solvent-dependent constants with *B *≈ 1, *v_o _*is the occupied volume, and *v_f _*is the free volume. The free volume is the temperature-dependent factor, and for glass-forming liquids, the free volume reaches a minimum at the glass transition temperature [66]. The ratio *v_f _*/*v_o _*is the relative free space for a liquid and becomes very small, typically 0.025, at the glass transition of many alcohols. Loutfy and Arnold [63] provide experimental evidence that the quantum yield of a fluorophore follows a relationship analogous to Equation 21,

(22) $$\phi_{F}=\frac{k_{R}}{k_{NR,0}}\cdot exp\left(x\frac{v_{o}}{v_{f}}\right)$$

where *k*_*NR*,0 _is interpreted as an intrinsic, fluorophore-dependent constant, and *x *is the slope found in plots of *logϕ_F _*over $v_{f}^{-1}$. Equation 22 allows to express the rotational relaxation rate as a function of free volume, namely, *k_or _*= *k*_*NR*,0 _*exp*(-*x v_v_*/*v_f _*). Contrary to the assumptions by Vogel and Rettig and by Förster and Hoffmann, Loutfy and Arnold found 15 a power-law relationship between viscosity and diffusional reorientation rate. Equation 21 can be used to replace the free-volume term by viscosity, which yields Equation 23:

(23) $$\phi_{F}=\frac{k_{R}}{k_{NR,0}}{\left(\frac{\eta }{A}\right)}^{x}=C\cdot \eta^{x}$$

By combining the dye- and solvent-dependent constants *k_R_*, *k*_*N R*,0_, and *A^x ^*into one constant *C*, Equation 1 readily emerges. It is noteworthy that the rigorous derivation by Förster and Hoffmann - under the assumption of rotational friction according to the DSE model - and the more empirical derivation by Loutfy and Arnold - under the assumption of a power-law microfriction behavior - lead to the same relationship between quantum yield and bulk viscosity. Contrary to the Förster- Hoffmann derivation, however, the exponent *x *in Equation 23 can vary with the solvent and the molecular rotor molecule.

**Figure 3:**
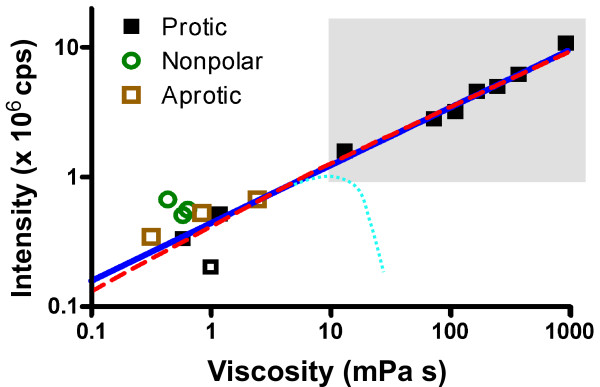
**Comparison of the models for the quantum yield - viscosity relationship**. Shown is the peak emission intensity of 5 *μ*M DCVJ in various solvents. Black squares indicate polar, protic solvents (single- and polyfunctional alcohols); green circles indicate dipolar, aprotic solvents, and brown squares indicate nonpolar solvents. Equations 1 and 20 have been fitted to the intensity/viscosity data of the polar solvents (black squares) with the exception of water (open square). The gray region indicates a viscosity gradient made of mixtures of ethylene glycol and glycerol. Equation 1 creates a straight line (blue) in the double-logarithmic plot with a slope of *x *= 0.45, whereas Equation 20 (red) curves and drops below Equation 1 for large and small *η*. It can be seen that polarity effects dominate at low viscosities. The light-blue dotted line indicates qualitatively a deviation reported by Law [15] in long-chained 1-alkanols.

In practice, each of the models has limited applicability. A comparison of the model by Vogel and Rettig to the model by Loutfy *et al*. is shown in Figure 3. Each data point represents solvent viscosity and measured intensity of the molecular rotor DCVJ at a concentration of 5 *μ*M. Intensity is proportional to quantum yield, and an additional proportionality constant needs to be introduced in Equations 1 and 20 to reflect concentration and instrument constants. It can be seen that Equation 1 describes the data from polar solvents well in accordance with the literature [15,34,40,54]. Equation 20 describes the data almost equally well, although Equation 1 is statistically the preferred model (F-test, *P *= 0.89). The model in Equation 20 tends to underestimate the viscosity at very low and very high solvent viscosities. In fact, when *A *≪ *B*, Equation 20 takes up the form of Equation 1. The curve fit in Figure 3 provided *A ≈ B*/10. However, this ratio is not in agreement with values reported by Vogel and Rettig, where *A *and *B *are in the same order of magnitude. In this example, high viscosities were achieved with a viscosity gradient of mixtures of glycerol and a low-viscosity alcohol, such as ethylene glycol or methanol. This method is commonly used in the literature [33,40,54,56,63,67] to achieve large-scale variations in viscosity with relatively small variations in polarity.

Deviations from the model can be seen in several instances. Water with its very high polarity reduces the barrier to the TICT state [50] and causes an anomalous low fluorescence. Polar aprotic solvents, such as dimethylsulfoxide, dimethylformamide, and acetone show a higher DCVJ intensity than predicted by the models, and nonpolar solvents (methylene chloride, benzene and toluene) have an even higher intensity, because nonpolar solvents stabilize the LE state. It can be seen that at low viscosities, other effects than microfriction dominate. Law [15] has reported that the chain length of short-chain 1-alkanols has a very small effect on the quantum yield, which would corroborate the low viscosity case presented by Förster and Hoffmann (Equation 7). According to Law [15], long-chain 1-alkanols also deviate from the models (light blue dotted line in Figure 3), because the alkane chain becomes the main determinant of intramolecular rotation, and the viscosity of alkanes is known to be much lower than that of the corresponding 1-alkanols.

In summary, rotational diffusivity is the most important determinant of intramolecular rotation rate and therefore a molecular rotor's quantum yield. However, when the intramolecular rotation rate becomes very high in solvents of low viscosity, additional effects, such as hydrogen bond formation, excimer formation, and polar-polar interaction are no longer negligible and cause significant deviations from established models that describe the relationship between quantum yield and viscosity.

## Engineering Aspects of Molecular Viscometers

There is a growing need for viscosity reporters with microscopic resolution and ultrafast response. Due to the nature of the twisted-state formation, which takes place within tens of picoseconds [68], a molecular rotor reports changes in the local microviscosity almost instantaneously. Since molecular rotors are affected only by their immediate microenvironment, they can be used to report spatially resolved microviscosity with resolution limited only by the optical equipment. These two features explain the popularity of molecular rotors in cell and vesicle research [40,43-45,69-73]. Furthermore, molecular rotors enjoy high popularity as real-time probes of polymerization processes [62,74,75], where one reason is the poor suitability of conventional methods due to their invasive nature and associated destructiveness, and their poor accuracy [76]. Conversely, molecular rotors allow *in situ *probing. Other potential areas of application are food science, for example, the crystallization behavior of lactose [77] or the behavior of soy flour according to the Williams-Landel-Ferry model [78], and the measurement of bulk viscosity of biofluids, where short measurement and turnaround times may accelerate serial viscosity measurement by orders of magnitude [26]. The key to these applications is the potential of a quantitatively accurate measurement.

### Steady-State Spectroscopy and Intensity Measurements

Emission intensity *I_EM _*and quantum yield *ϕ_F _*are proportional. Therefore, steady-state fluorescence spectroscopy can be calibrated to provide quantum yield from peak spectral intensity. In any steady-state instrument, Equation 24 holds for low dye concentrations:

(24) $$\begin{array}{l} I_{EM}=G\cdot c\cdot I_{EX}\cdot \phi_{F}\\ =\left(G\cdot c\cdot I_{EX}\cdot C\right)\cdot \eta^{x} \end{array}$$

Here, *G *is the instrument gain factor, *c *is the dye concentration, and *I_EM _*is the emission light intensity. If *ϕ_F _*is substituted with Equation 1, these factors are combined together with the dye-dependent constant *C*, into one calibration constant that can be determined with reference fluids. This calibration process is comparable to that of mechanical rheometers, although the exponent *x *plays an important role and needs to be determined beforehand for each specific class of fluids, such as alcohols, aqueous solutions, or oils. If the proportionality factors in Equation 24 are known, they can be combined into a single calibration constant *ξ*, and the equation can be solved for *η*:

(25) $$\eta =\xi \cdot {\left(I_{EM}\right)}^{\frac{1}{x}}$$

With this method the precision of mechanical rheometers can be reached or exceeded [26]. However, intensity measurements can be confounded if the solvent is absorbent (colored) or scattering. Spectrofluorometers exist that can simultaneously measure fluorescence emission and spectral absorption. In practice, it is difficult to distinguish dye absorption from fluid absorption, but dye concentration and its absorption coefficient are usually known. In this case, it is possible to correct the measured intensity and obtain a value that represents the idealized intensity in the absence of fluid absorption. The fraction of excitation light *I_A _*that passes through the sample can be described by Beer's law as *I_A _*= *I*_0 _*exp*(-ϵ*cl*) where ϵ is the dye extinction coefficient and *l *is the length of the light path though the sample. The difference between the incident light *I*_0 _and the exiting light *I_A _*is available for dye excitation. Measurement of *I_A _*allows to calculate one unknown coefficient, either *c *or *l*. The corrected *I_EX _*can be used in Equation 24. Although this method can be used to reduce the influence of variations in dye concentration in a clear fluid, its practical relevance becomes much higher in absorbing or scattering fluids [28]. Examples are blood plasma and lymphatic fluid, which preferentially absorb blue light and solutions of macromolecules, which often exhibit Rayleigh scattering. If a solvent has an absorption coefficient *μ_A_*, less light is available for photoexcitation, and *I_EX _*can be approximated with Equation 26:

(26) $$I_{EX}=I_{0}\frac{\epsilon cl}{\mu_{A}+\epsilon cl}\cdot \left(1-e^{-\left(\mu_{A}+\epsilon c\right)l}\right)$$

**Figure 4:**
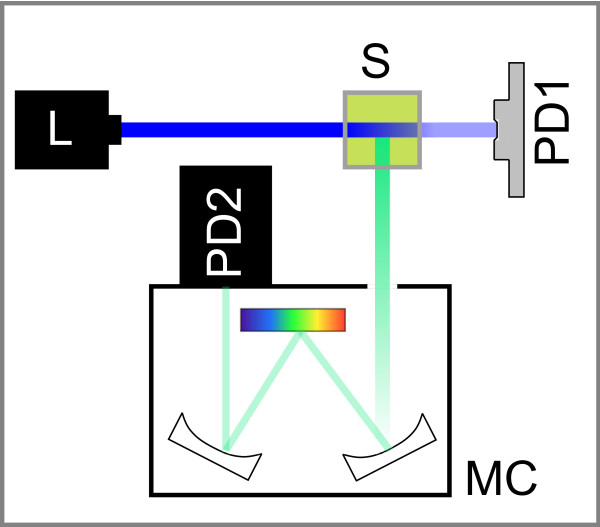
**Proposed spectrophotometer with the capability to measure fluorescence intensity, absorbance, and scattering simultaneously**. The excitation light source **L **can be a laser, a highly collimated light-emitting diode (LED), or a broadband source with selectable excitation filters. Excitation light is directed onto the sample **S**. Light that is not absorbed by the sample reaches the photodetector **PD1**, which can be a photodiode or a photomultiplier tube. The measurement of absorbed light against a blank sample provides total sample absorbance. Fluorescence emission is collected in a monochromator **MC **and can be spectrally resolved and analyzed in the second detector **PD2**, which needs to be a photomultiplier tube. Single-wavelength measurements are possible by using filters instead of a monochromator. Fluorescence collection uses the conventional L geometry, because the smallest amount of scattered light is found perpendicular to the excitation beam. However, a scattering sample causes a measurable amount of excitation light to be scattered into the monochromator, and by selecting the excitation wavelength, the amount of scattering can be determined. This device is similar to the one presented by Milich *et al*. [28].

Again, absorption measurement can provide the unknown *μ_A_*, and when the dye concentration is known, a corrected excitation intensity can be computed for Equation 24. A spectrophotometer that measures fluorescence, scattering, and absorbance simultaneously can be designed with relatively few, low-cost components. One possible design is shown in Figure 4. A single light source provides excitation light for the sample. Absorbance is measured with a photodiode, and both scattering and fluorescence intensity are measured with a photomultiplier through a monochromator.

Correction for fluid optical properties becomes even more complex when the dye concentration is high and when the solvent absorption is strongly wavelength-dependent. The presence of scatterers further complicates the correction. In two relatively simple cases of forward-scattering microspheres and of starch solutions, the average excitation path length was found to be increased, and the presence of the scatterer increased fluorescence intensity. By measuring the scattering intensity, a corrected fluorescence emission was found that almost completely eliminated the influence of the scattering agent [28]. Higher scatterer content, however, would again reduce the measured intensity, and additional studies need to be performed to obtain correction formulas or algorithms for different types and concentrations of scatterers, and for combinations of scatterers and absorbers.

### Time-Resolved Spectroscopy

Time-resolved spectroscopy promises to overcome some of the challenges associated with steady-state measurements. Most important, quantum yield and lifetime *τ *are directly related without the proportionality factors seen in Equation 24. When the natural lifetime *τ*_0 _- the largest possible lifetime for a fluorphore - is known, for example from a measurement in supercooled glass, the quantum yield can be computed as *ϕ_F _*= *τ*/*τ*_0_. The resulting quantum yield can be used directly in Equation 1, which leads to the lifetime equation analogous to Equation 25:

(27) $$\eta ={\left(\frac{\tau }{\tau_{0}C}\right)}^{\frac{1}{x}}$$

Natural lifetimes for some molecular rotors have been found to be between 3 ns and 4 ns [15,64], and actual lifetimes can be in the low picosecond range for low-viscosity solvents [64]. To accurately measure such short lifetimes, relatively sophisticated instruments are required, which can be very expensive compared to simple steady-state instruments.

Moreover, lifetime measurements may reveal multiexponential decay behavior. Vogel and Rettig [15] found double-exponential decays in triphenylmethane dyes and attributed the two decay components to DSE and free-volume diffusion, respectively. Law [15] reported that solvent diffusional rotation causes shorter measured lifetimes. Multiexponential relaxation dynamics that were dominated by solvent relaxation constants were also found by Dutta and Bhattacharyya [79], who reported lifetime constants in the low picosecond range and in the low nanosecond range, whereby the nanosecond range carried significant information about the type of solvent. Hara *et al*. [80] found triple-exponential decay functions when they applied pressure to the solvents to cause a pressure-induced viscosity increase. The analysis of lifetime experiments is complex, because many levels of solvent-rotor interaction, such as diffusion, electrostatic and polar interaction, and hydrogen bonding influence the lifetime dynamics and lead to complex decay patterns. This level of complexity cannot be seen in steady-state experiments. Whereas steady-state measurements can be confounded by solvent- and instrument-related factors, lifetime experiments are affected by the complex rotor-solvent interaction. More research is needed to separate and interpret the lifetime components and find an accurate relationship to the solvent's microviscosity.

On the other hand, spatially-resolved lifetime measurement, for example, fluorescence lifetime imaging microscopy (FLIM) [81] is a promising method, and in many cases a simplified lifetime-viscosity relationship (Equation 27) is sufficiently accurate for physiological studies. The major advantage of FLIM over steady-state fluorescence microscopy is its in-dependence from local dye concentration gradients, which makes FLIM an ideal method for studies in living cells [56,57]. With the exception of very expensive and advanced devices, FLIM is limited to single-exponential decays [81], and the complexity of the decay dynamics described in the previous section is difficult to reproduce.

### Ratiometric Measurements and Self-Calibrating Dyes

**Figure 5:**
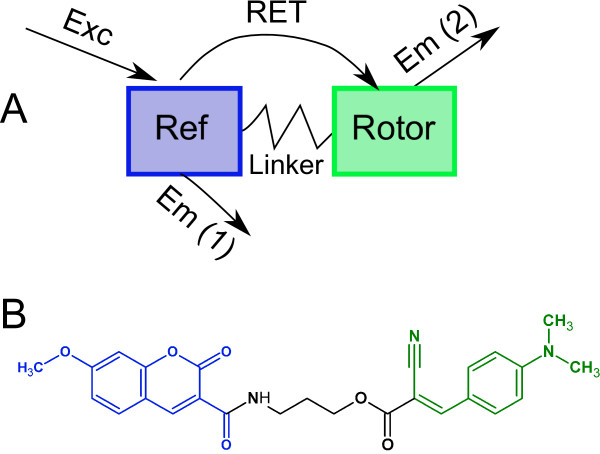
**Basic structure of an engineered ratiometric dye system (A)**. A conventional fluorescent molecule that is not viscosity-sensitive serves as intensity reference (Ref) and is coupled to a molecular rotor through a linker structure. The fluorescent groups can be excited individually and emit fluorescence at their typical wavelength. However, when the reference unit is excited, it transfers some of its excited-state energy to the rotor via resonance energy transfer (RET), and dual emission from both reference and rotor can be observed. One possible chemical structure is shown in B. The reference is a coumarin (blue), and the rotor is a structure is based on an aniline motif rather than the tricyclic julolidine found in DCVJ.

**Figure 6:**
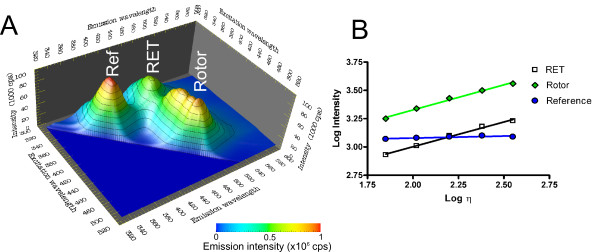
**Emission profile of the ratiometric dye system in Figure 5**. The fluorescence fingerprint (**A**) shows the emission intensity as a function of both excitation and emission wavelength. Three distinct emission peaks emerge. The first is caused by the reference fluorophore (**Ref**), the second by the molecular rotor where the excitation of the rotor molecule takes place through resonance energy transfer from the reference (**RET**), and the third is emitted from the rotor after direct excitation of the rotor (**ROTOR**). **B **shows the emission maxima in solvents of different viscosity. It can be seen that the reference emission is widely independent of the viscosity, and small variations can be attributed to refractive index changes. Conversely, rotor emission increases with viscosity in a power-law fashion, irrespective of whether the rotor is excited directly or through RET.

A third approach to reduce the influence of local concentration gradients and some sample optical properties (absorption, scattering) are engineered ratiometric dye systems [82]. It is possible to covalently couple a molecular rotor to a reference fluorophore that is not viscosity-sensitive (Figure 5). The two covalently linked fluorophores exhibit three distinct emission peaks as shown in Figure 6: direct emission from the reference fluorophore, direct emission form the molecular rotor, and indirect emission from the molecular rotor, where excited-state energy is transferred from the reference fluorophore through resonance energy transfer (RET). Since the two units are covalently linked, their local concentration is always identical. Therefore, the instrument-dependent constants *G *and *I_EX _*and the concentration *c *cancel out in Equation 24 when the ratio of rotor emission to reference emission is used. The emission light of both fluorophores also experiences the same absorption and scattering. The ratiometric method offers similar advantages as lifetime measurement, albeit with relatively low-cost steady-state equipment. One limitation occurs when fluid absorption and scattering become wavelength-dependent, in which case reference and rotor intensity need to undergo additional correction steps. Moreover, fluorescence intensity from RET is inherently less efficient than direct excitation. Particularly in low-viscosity cases, this method may suffer from a poor signal-to-background ratio in absorbing or scattering media. However, the spectra in Figure 6B demonstrate that intensity from RET is only about a factor 2 lower than direct intensity, which should be sufficient for most applications.

## Conclusion

Molecular rotors are a twisted-state-forming subgroup of intramolecular charge transfer fluorophores. The twisted-state formation rate is strongly dependent on the environment, most dominantly on the local diffusion coefficient. Two overarching groups of these fluorophores are those where relaxation from both LE and twisted states are associated with photon emission, and those where relaxation from the twisted state occurs radiationless. The former show a dual- band emission with a strong polarity-dependent solvatochromic shift and very strong dependency of the twisted-state emission band (the red-shifted band) on both polarity and viscosity of the medium. The latter show single-band emission from the LE state with a highly viscosity-dependent quantum yield. The viscosity-dependent emission is hypothesized to be related to rotational diffusion, although different theoretical treatments of the viscosity-dependence exist. Apart from viscosity, solvent polarity, hydrogen bond formation and excimer formation also play a role in the spectroscopic properties of molecular rotors. These complex interactions with the environment provide one impediment to using molecular rotors as fluorescent microrheometers. However, at viscosities above 2 mPa s, steric hindrance dominates twisted-state formation, and viscosity becomes the singularly most dominant factor to influence the molecular rotor's quantum yield. A power- law relationship between quantum yield and viscosity is most widely used, and this relationship is confirmed by experimental observation over more than three orders of magnitude of solvent viscosity. With molecular rotors, viscosity measurement can be reduced to either intensity measurements or fluorescent lifetime measurements. A particular strength of the fluorescent method lies in the ability to spatially resolve the emission (fluorescent imaging) with applications in biology, cell physiology and polymer chemistry. However, there are confounding factors that deserve further research. First, the optical properties of the microenvironment can influence the emission signal, and the emission needs to be corrected for absorption and scattering to obtain accurate microviscosity information. Second, the constants in the power-law relationship between quantum yield and viscosity require calibration for each fluid type. Most notably, the exponent × was found to be a constant of *x *= 2/3 by Förster and Hoffmann, but can be variable according to the free-volume theory by Loutfy *et al*. Further research into the rotor-solvent interaction will likely illuminate the constants used in the relationship between quantum yield and viscosity and therefore increase the accuracy with which the microvisocsity can be measured with molecular rotors.

## Competing interests

The authors declare that they have no competing interests.

## Authors' contributions

MH is responsible for the overall content and contributed the engineering aspects. ET contributed the chemistry-related content of this review. All authors have read and approved the final manuscript.

## Acknowledgements

The authors gratefully acknowledge research support from the National Institutes of Health through NIH grant 1R21 RR 025358.
